# Beyond the MRI-positive/MRI-negative dichotomy: A stepwise surgical strategy for Cushing's disease

**DOI:** 10.1016/j.bas.2026.106668

**Published:** 2026-09-05

**Authors:** Luca Ferlendis, Hung Thanh Chu, Thibault Passeri, Chiara Villa, Delphine Leclercq, Maxime Barat, Arianna Fava, Kevin Beccaria, Guillaume Assié, Sébastien Froelich, Bertrand Baussart

**Affiliations:** a Department of Neurosurgery, Lariboisière Hospital, Assistance Publique – Hopitaux de Paris, University of Paris, Paris, 75010, France; b Division of Neurosurgery, Department of Medical and Surgical Specialties, Radiological Sciences and Public Health, University of Brescia, ASST Spedali Civili, Brescia, Italy; c Surgery Department, Hanoi Medical University, Hanoi, Viet Nam; d Department of Pathology, Lariboisière Hospital, Assistance Publique-Hôpitaux de Paris, Paris, 75010, France; e Université Paris Cité, CNRS, INSERM, Institut Cochin, Paris, F-75014, France; f Department of Functional and Diagnostic Neuroradiology, AP-HP, Pitié-Salpêtrière Hospital, Paris, 75013, France; g Department of Radiology, Hôpital Cochin, Assistance Publique-Hôpitaux de Paris, Paris, 75014, France; h Université Paris Cité, Faculté de Médecine, Paris, 75006, France; i Department of Pediatric Neurosurgery, Necker Hospital, Assistance Publique-Hôpitaux de Paris, Paris, 75006, France; j Department of Endocrinology, Hôpital Cochin, Reference and Competence Center for Rare 49 Adrenal Diseases and Rare Pituitary Diseases, Assistance Publique-Hôpitaux de Paris, Paris, 75014, France

**Keywords:** Cushing disease, Pituitary surgery, Spectrum, Endoscopic endonasal, Mononostril, MRI-negative

## Abstract

**Introduction:**

Cushing's disease (CD) has traditionally been approached through a binary paradigm based on MRI findings, distinguishing MRI-positive from MRI-negative cases. However, this dichotomy incompletely reflects the heterogeneity of corticotroph tumors and complexity of intraoperative decision-making.

**Research question:**

We propose a stepwise surgical strategy that moves beyond this binary stratification by conceptualizing CD as a spectrum integrating imaging, biochemical, and intraoperative findings.

**Methods:**

This retrospective purposefully selected illustrative case series was drawn from a prospective database of 983 patients treated for CD between 2006 and 2025. All underwent mononostril endoscopic transsphenoidal surgery by an expert pituitary surgical team. Seven cases were chosen to illustrate the CD spectrum presentations, including MRI-positive lesions with invasive features, MRI-equivocal findings, and MRI-negative disease. A stepwise algorithm integrating preoperative imaging, functional analysis, and intraoperative findings guided surgical exploration and resection.

**Results:**

Seven patients were included. In MRI-visible lesions, targeted adenomectomy was performed, with extension to basal dura or cavernous sinus wall when indicated. In MRI-negative or equivocal cases, exploration was guided by repeat imaging, heterogeneous MRI signal, venous sampling, and/or FET-PET. When tumor tissue was identified, selective or enlarged adenomectomy was performed according to infiltration patterns. Early endocrine remission was achieved in 6 patients, while one patient achieved postoperative eucortisolism. Transient diabetes insipidus was the only postoperative complication.

**Conclusion:**

CD may be reconsidered beyond a binary MRI-based paradigm, as a spectrum of scenarios. A stepwise surgical strategy better reflects disease heterogeneity and provides a practical framework for surgical decision-making, particularly in MRI-negative or MRI-equivocal cases.

## Highlights

• Cushing's disease may be better conceptualized as a continuum rather than MRI-positive/MRI-negative categories.
• A stepwise surgical framework integrates dynamic preoperative and intraoperative findings.
• Repeat imaging and medical control can reveal actionable surgical targets over time.
• Individualized surgical strategy is key to optimize endocrine outcomes.
• This spectrum-based approach provides practical guidance for surgical decision-making.

## Abbreviations:

ACTH: adrenocorticotropic hormone
BD: basal dura
CD: Cushing's disease
CR: clival recess
CS: cavernous sinus
DI: diabetes insipidus
EoR: extent of resection
FET-PET: fluoroethyl-tyrosine positron emission tomography
ICA: internal carotid artery
IPSS: inferior petrosal sinus sampling
MRI: magnetic resonance imaging
MWCS: medial wall of the cavernous sinus
PA: pituitary adenoma
PDS: polydioxanone
PG: pituitary gland
PitNET: pituitary neuroendocrine tumor
QoL, quality of life
TS: tuberculum sellae

## 1 Introduction

Cushing's disease (CD) remains a major diagnostic and surgical challenge, particularly in patients without a clear radiological target. Corticotroph adenomas/PitNETs are microadenomas in approximately 90% of cases (Reincke and Fleseriu, 2023). Despite advances in imaging, no lesion is identifiable on preoperative MRI in 23-50% of patients (Reincke and Fleseriu, 2023; Fleseriu et al., 2021; Ghidoni et al., 2026), even with dynamic sequences or high-resolution 3T MRI. Remission rates in MRI-negative cases are slightly lower than in MRI-positive cases (approximately 68–75% vs 77–85%)(Ghidoni et al., 2026; Cristante et al., 2019; Shapey et al., 2026; Dai et al., 2020)–(Ghidoni et al., 2026; Cristante et al., 2019; Shapey et al., 2026; Dai et al., 2020). In MRI-negative cases, surgery remains the first-line treatment in the absence of effective alternatives (Cristante et al., 2019; Zhang et al., 2020), provided that the diagnosis is confirmed by clinical, biochemical, and additional investigations, including inferior petrosal sinus sampling (IPSS)(Fleseriu et al., 2021; Cristante et al., 2019; Yang et al., 2022).Importantly, recurrence also remains a major concern, with rates ranging from 10% to 30% after initial remission (Shapey et al., 2026; Gadelha et al., 2023), reinforcing the need for optimized strategies.

Current clinical practice has traditionally been structured around a binary paradigm distinguishing MRI-positive from MRI-negative CD. While useful for diagnostic categorization, this dichotomy fails to capture the heterogeneity of corticotroph tumors and the complexity of intraoperative decision-making, in which an individualized approach is increasingly advocated (Baussart et al., 2025a).

Advances in imaging techniques, including repeated high-resolution MRI, and improved biochemical control with modern steroidogenesis inhibitors (e.g. osilodrostat)(Gadelha et al., 2022), allow for a more refined characterization of pituitary lesions over time. Notably, in some patients, initially negative MRI findings later become equivocal or clearly lesion-positive, while subtle intrasellar heterogeneities may represent true surgical targets (Reincke and Fleseriu, 2023). Venous sampling techniques reliably confirm pituitary ACTH hypersecretion and may assist in tumor lateralization, although some discrepancies have been reported (Fleseriu et al., 2021; Ghidoni et al., 2026).

These observations support considering CD as a spectrum of clinical, radiological and functional presentations rather than a strictly binary entity. Within this continuum framework, surgical decision-making becomes dynamic, integrating evolving preoperative data with intraoperative findings. However, few studies have systematically described how this integration can be operationalized during surgery, particularly in MRI-negative and MRI-equivocal CD^8^.

Based on our experience as a high-volume center managing more than 50 CD cases per year (Baussart and Gaillard, 2021; Baussart et al., 2022; Baussart et al., 2025a; Cebula et al., 2017; Garvayo et al., 2023; Benanteur et al.; Baussart et al., 2025b; Wang et al., 2019; Passeri et al., 2026), we propose a structured, stepwise surgical strategy that conceptualizes CD as a spectrum rather than a binary condition. Through representative clinical cases and intraoperative video illustrations, we aim to provide a practical framework to guide surgical exploration and decision-making.

## 2 Material and methods

### 2.1 Patient selection

**Fig. 1:**
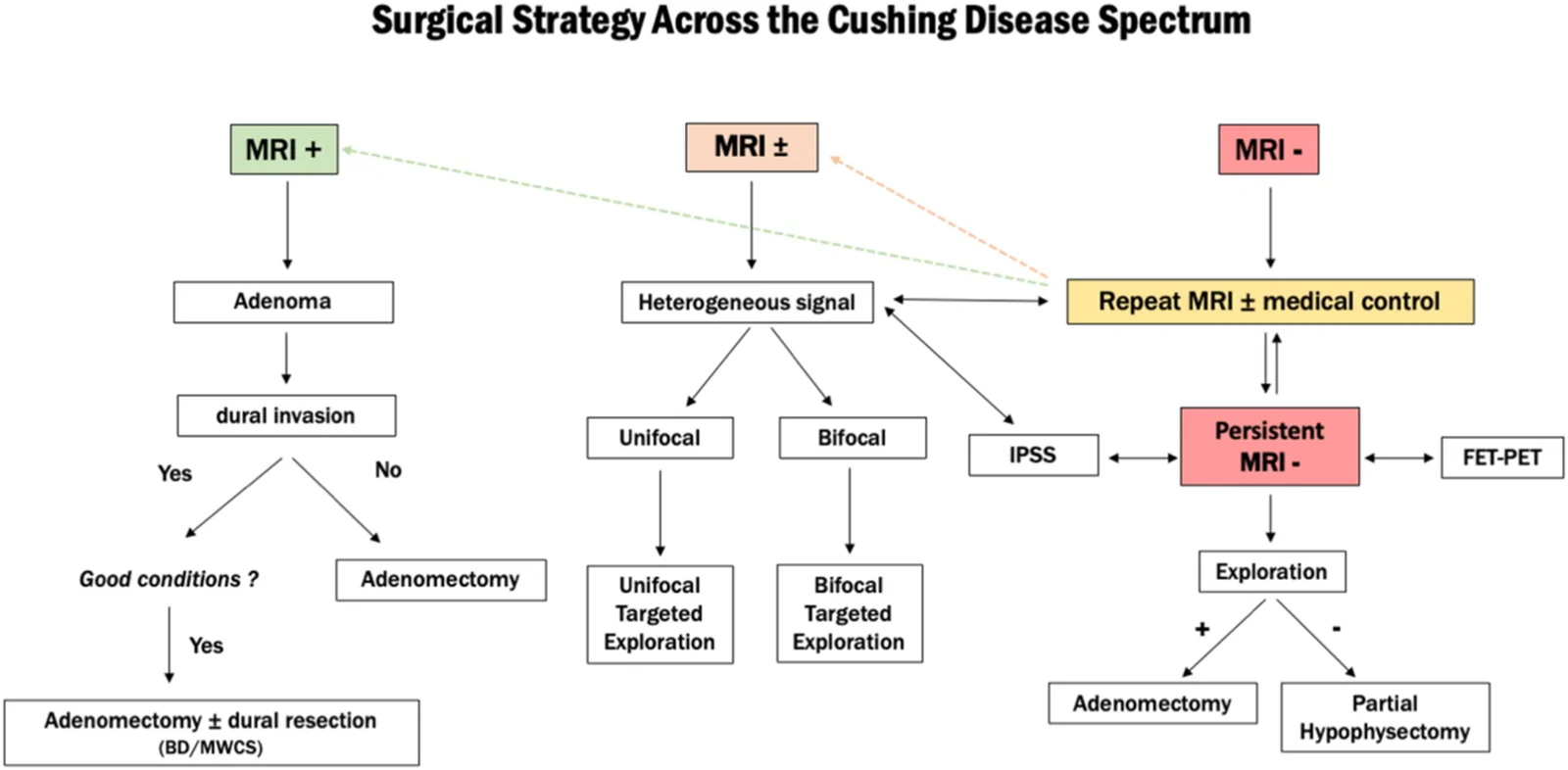
**Surgical algorithm for Cushing's disease across the MRI spectrum.** Management integrates MRI findings, repeat imaging, and, when indicated, IPSS, with surgical exploration and extent of resection determined according to radiological and intraoperative findings. *Abbreviations: BD: basal dura; IPSS: inferior petrosal sinus sampling; MWCS: medial wall of the cavernous sinus*.

This study is a retrospective, purposefully selected illustrative case series drawn from a prospectively maintained database. The database included 983 patients operated on for Cushing's disease between 2006 and 2025 by a single expert surgical team (B.B., S.G.). For the present report, seven non-consecutive patients with challenging corticotroph adenomas were selected, including cases with MRI-positive, MRI-equivocal, and MRI-negative findings. Cases were selected by the expert pituitary team after outcomes were known, based on their educational value and ability to illustrate the different clinical, radiological, and surgical scenarios represented in our current decision-making algorithm (Fig. 1).

All patients had a confirmed clinical and biochemical diagnosis of CD. Surgical indications and timing were systematically discussed within a multidisciplinary pituitary tumor board involving neurosurgeons, endocrinologists, and neuroradiologists. Histological analysis confirmed the diagnosis of CD based on transcription factor expression (T-PIT positivity) and immunohistochemical staining for pituitary hormones (ACTH positivity), except in case 5, where pathology was negative.

### 2.2 Surgical technique

All patients were treated using a mononostril endoscopic transsphenoidal approach, using the surgical technique previously detailed (Baussart and Gaillard, 2021; Baussart et al., 2021). After large sphenoidotomy and tailored sellar floor opening, the dura was usually incised in an inverted U-shaped fashion to allow full exposure of the pituitary gland (Baussart et al., 2025a). The inferior intercavernous sinus was coagulated and sectioned when necessary to improve visualization and access to the inferior gland.

Intraoperative tumor identification was based on color and consistency, compared to normal gland tissue. The extent of tumor resection was determined according to intraoperative findings: (i) well-circumscribed lesions were treated by selective adenomectomy; (ii) poorly defined lesions required an enlarged adenomectomy including a rim of junctional pituitary tissue.

Sellar floor reconstruction was performed according to the principles previously described (Baussart et al., 2022, 2024). Whenever feasible, we reconstructed the sellar floor with an autologous bone graft or with a synthetic polydioxanone (PDS) plate.

### 2.3 Surgical algorithm for pituitary exploration

Our surgical management has progressively evolved over the 20-year period, and Fig. 1 represents our current decision-making algorithm. All seven illustrative cases included in this study were operated between 2024 and 2025 and were managed according to this current strategy.

Adenomectomy was defined as selective when limited to the identified tumor and as enlarged when extended to the adjacent junctional pituitary tissue. Partial hypophysectomy was defined as resection of the inferior third of the pituitary gland, including anterior and posterior components, when no tumor was identified after systematic exploration.

IPSS was indicated in MRI-equivocal or MRI-negative cases, with the primary goal to confirm a pituitary source of ACTH secretion. When IPSS lateralization was available, it was considered supportive in determining the initial side of exploration.

In MRI-positive cases, defined by the presence of a clearly identifiable surgical target, targeted adenomectomy was performed, either selective or enlarged to the gland when macroscopic infiltration was identified. When intraoperative evidence of dural invasion was present, an extended resection was usually performed. In cases of macroscopic basal dura invasion, the dural flap was resected and extended inferiorly to improve completeness of resection, as previously described (Baussart et al., 2025a) (**Video 1**). The borders of the dural defect were coagulated to optimize local control. In selected patients with cavernous sinus invasion, the medial wall of the cavernous sinus was resected, and the intracavernous tumor component was removed when feasible. Adequate operative conditions and clear visualization of the parasellar anatomical landmarks are essential. Hemostatic agents to control venous bleeding represent an important technical step in achieving these conditions.

MRI-equivocal cases were defined by the presence of a focal or bifocal signal abnormality on T1-or T2-weighted sequences, confirmed on repeat imaging, and considered a potential but uncertain surgical target. In these patients, targeted exploration of the identified areas was performed.

MRI-negative cases were defined by the absence of an identifiable or equivocal surgical target on at least two dedicated pituitary MRI examinations reviewed by an experienced neuroradiologist. During the preoperative period, some patients received steroidogenesis inhibitors (e.g., ketoconazole, metyrapone, or more recently osilodrostat) to control hypercortisolism. Intraoperatively, the pituitary gland was incised horizontally and systematically explored using small curettes. If no tumor was identified on the initial side, the contralateral side was systematically explored. If no lesion was identified after bilateral exploration, partial hypophysectomy was performed in selected patients with uncontrolled hypercortisolism (Cebula et al., 2017). Given that a substantial proportion of corticotroph tumors may arise from the neurohypophysis (Cebula et al., 2017; Weil et al., 2006), both anterior and posterior components were included in the resection.

### 2.4 Illustrative cases

Seven representative cases were selected to illustrate the spectrum of CD presentations (Fig. 1), beyond the MRI-positive/MRI-negative dichotomy:

1. MRI-positive with a paracavernous microadenoma
2. MRI-positive with a microadenoma and basal dura invasion
3. MRI-positive with a macroadenoma, basal dura and cavernous sinus invasion
4. MRI-positive with a microadenoma and cavernous sinus invasion
5. MRI-equivocal with a unifocal heterogeneous signal
6. MRI-equivocal with a bifocal heterogeneous signal
7. MRI-negative with intraoperative adenoma identification

These cases were chosen for their clinical value in surgical practice.

### 2.5 Postoperative care and outcome assessment

Patients received standard perioperative hydrocortisone supplementation (20 mg three times daily) from the day of surgery. The evening dose on postoperative day 1 was withheld, and serum cortisol was measured on postoperative day 2. Transfer to the endocrinology department typically occurred on postoperative day 3.

According to the current recommendations for Cushing's disease management (Reincke and Fleseriu, 2023; Fleseriu et al., 2021; Baussart et al., 2025a), remission was defined as: (i) improvement of clinical manifestations, and (ii) postoperative biochemical cortisol deficiency (serum cortisol <2–5 μg/dL). However, in some patients with obvious cavernous sinus invasion, surgical outcomes may also be considered favorable when resulting in postoperative eucortisolism without the need for additional therapy (Baussart et al., 2025a).

### 2.6 Ethic statement

All procedures were performed in accordance with institutional ethical standards. Informed consent for surgery and use of anonymized clinical and imaging data was obtained from all patients.

## 3 Results

Clinical and surgical data of the seven illustrative cases were summarized in Table 1. Seven patients (median age 41 years, range 13–53; 5 females) were included: four MRI-positive, two MRI-equivocal, and one MRI-negative. IPSS was performed in two patients and FET-PET in one. Complete resection was achieved in 6/7 patients, with histological confirmation in 6/7. Early endocrine remission was achieved in 6/7 patients, while one patient achieved postoperative eucortisolism. Transient diabetes insipidus occurred in one patient, with no permanent pituitary hormonal deficits, neurovascular injuries, or other medical complications. Median follow-up was 12 months (range 6–14).

**Table 1:** Summary of the seven selected patients illustrating challenging tumors across the spectrum of Cushing's disease.

Case	Sex, Age	MRI Spectrum	MRI Size	MRI Features	MRI Number before surgery	MRI Diameter (mm)	IPSS	FET-PET	Preop TT	YOS	Surgical strategy	EoR	Complications	Histology	Endocrine status	Follow-up (months)
**1**	F, 53	MRI+	Microad	No invasion	1	8	No	No	Yes	2024	s-Adenomectomy	Complete	No	Positive	Remission	12
**2**	F, 13	MRI+	Microad	BD invasion	3	6	No	No	Yes	2024	s-Adenomectomy + BD resection	Complete	Transient DI	Positive	Remission	14
**3**	F, 42	MRI+	Macroad	BD + CS invasion	1	11	No	No	No	2025	s-Adenomectomy + BD/CS resection	Subtotal	No	Positive	Eucortisolism	6
**4**	F, 41	MRI+	Microad	CS invasion	2	4	No	Yes	Yes	2025	e-Adenomectomy + CS resection	Complete	No	Positive	Remission	8
**5**	M, 13	MRI±	-	Unifocal signal	2	2	No	No	No	2024	Unifocal t-Exploration + e-Adenomectomy	Complete	No	Negative	Remission	13
**6**	M, 51	MRI±	-	Bifocal signal	3	3 (left)/3 (right)	Yes	No	No	2025	Bifocal t-Exploration + e-Adenomectomy	Complete	No	L: Positive R: CC	Remission	6
**7**	F, 37	MRI−	-	No target	3	-	Yes	No	Yes	2024	nt-Exploration + e-Adenomectomy	Complete	No	Positive	Remission	12

Abbreviations.

BD, basal dura; CS, cavernous sinus; DI, diabetes insipidus; e-Adenomectomy: enlarged adenomectomy; EoR, extent of resection; F, female; IPSS, inferior petrosal sinus sampling; Macroad, macroadenoma; M, male; Microad, microadenoma; MRI, magnetic resonance imaging; MRI±, MRI equivocal; MRI-, MRI-negative; MRI+, MRI-positive; nt-exploration: non-targeted exploration; s-Adenomectomy: selective adenomectomy; t-Exploration: targeted exploration; TT, medical therapy.

### 3.1 Case 1: MRI-positive with a paracavernous microadenoma

**Fig. 2:**
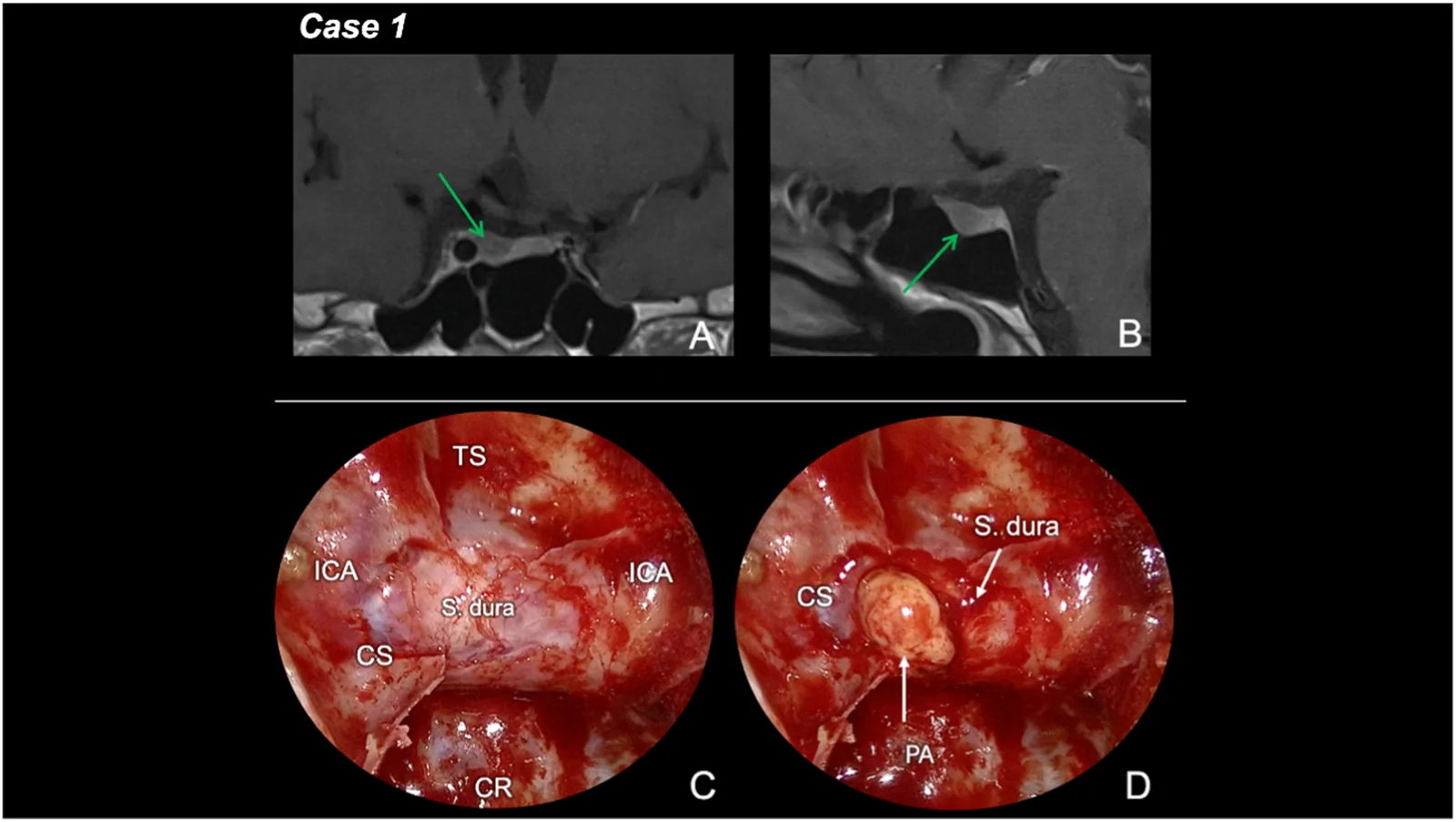
MRI-positive with a paracavernous microadenoma (Case 1) (A–B) Contrast-enhanced T1-weighted pituitary MRI showing an 8-mm right paracavernous ACTH-secreting microadenoma (green arrows). (C–D) Intraoperative endoscopic views demonstrating sellar opening, dural incision, and pituitary adenoma (PA) identification. *Abbreviations*: ICA: internal carotid artery; CS: cavernous sinus; S.dura: sellar dura; CR: clival recess; TS: tuberculum sellae.

A 53-year-old female presented with a 7-month history of CD. Preoperative MRI demonstrated a right paracavernous pituitary microadenoma (Fig. 2). Due to disease severity, partial biochemical control was achieved with osilodrostat prior to surgery.

The adenoma was clearly identified and selectively resected, with no obvious CS invasion (**Video 2**).

No postoperative complications occurred. Early biochemical remission was achieved, persisting at 12 months after surgery. Postoperative MRI at 3 months showed complete resection.

### 3.2 Case 2: MRI-positive with a microadenoma and basal dura invasion

**Fig. 3:**
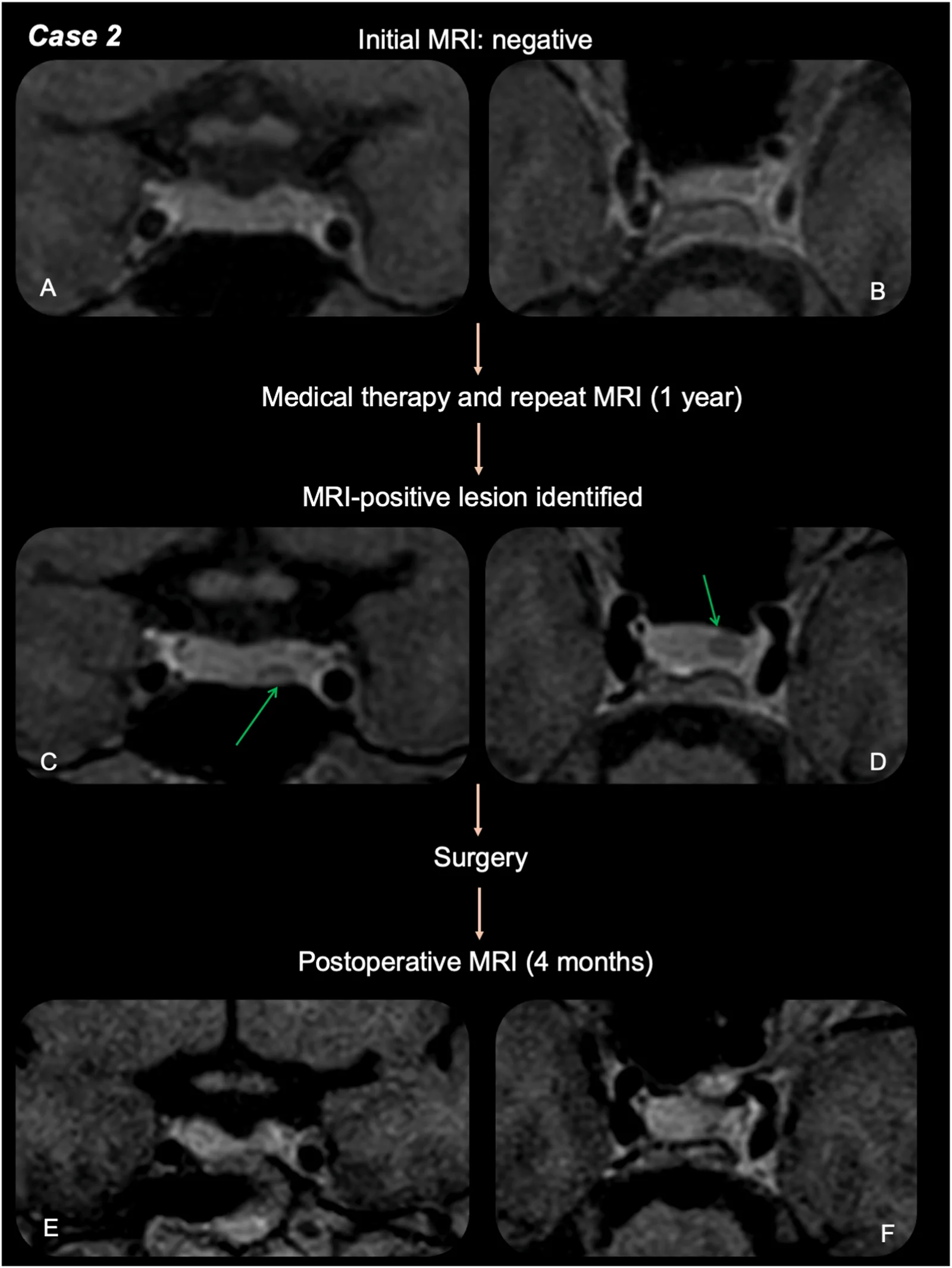
MRI-positive with a microadenoma and basal dura invasion (Case 2) (A–B) Initial coronal T1-weighted pituitary MRI showing no clear identifiable lesion. Following one year of medical therapy with osilodrostat and repeat imaging, a focal 6-mm left-sided lesion became visible. (C–D) Repeat MRI demonstrating a left-sided basal microadenoma (green arrows), consistent with an ACTH-secreting lesion. (E–F) Postoperative MRI at 4 months showing complete resection after endoscopic transsphenoidal adenomectomy with basal dura resection, with no residual tumor.

A 13-year-old female presented with a 16-month history of CD. Initial MRIs were considered negative, and medical therapy with osilodrostat was initiated. After one year of medical therapy, repeat MRI demonstrated a left basal microadenoma (Fig. 3).

An adenomectomy was performed and extended to the basal dura, according to the macroscopic appearance of dural infiltration.

Immediate transient diabetes insipidus occurred at day 1 and persisted for 5 days. Remission was maintained at the last follow-up examination, 14 months after surgery. Postoperative MRI confirmed complete resection.

### 3.3 Case 3: MRI-positive with a macroadenoma, basal dura and cavernous sinus invasion

A 42-year-old female presented with a 7-month history of CD. Preoperative MRI demonstrated a right-sided pituitary tumor with clear basal dura and cavernous sinus invasion.

**Fig. 4:**
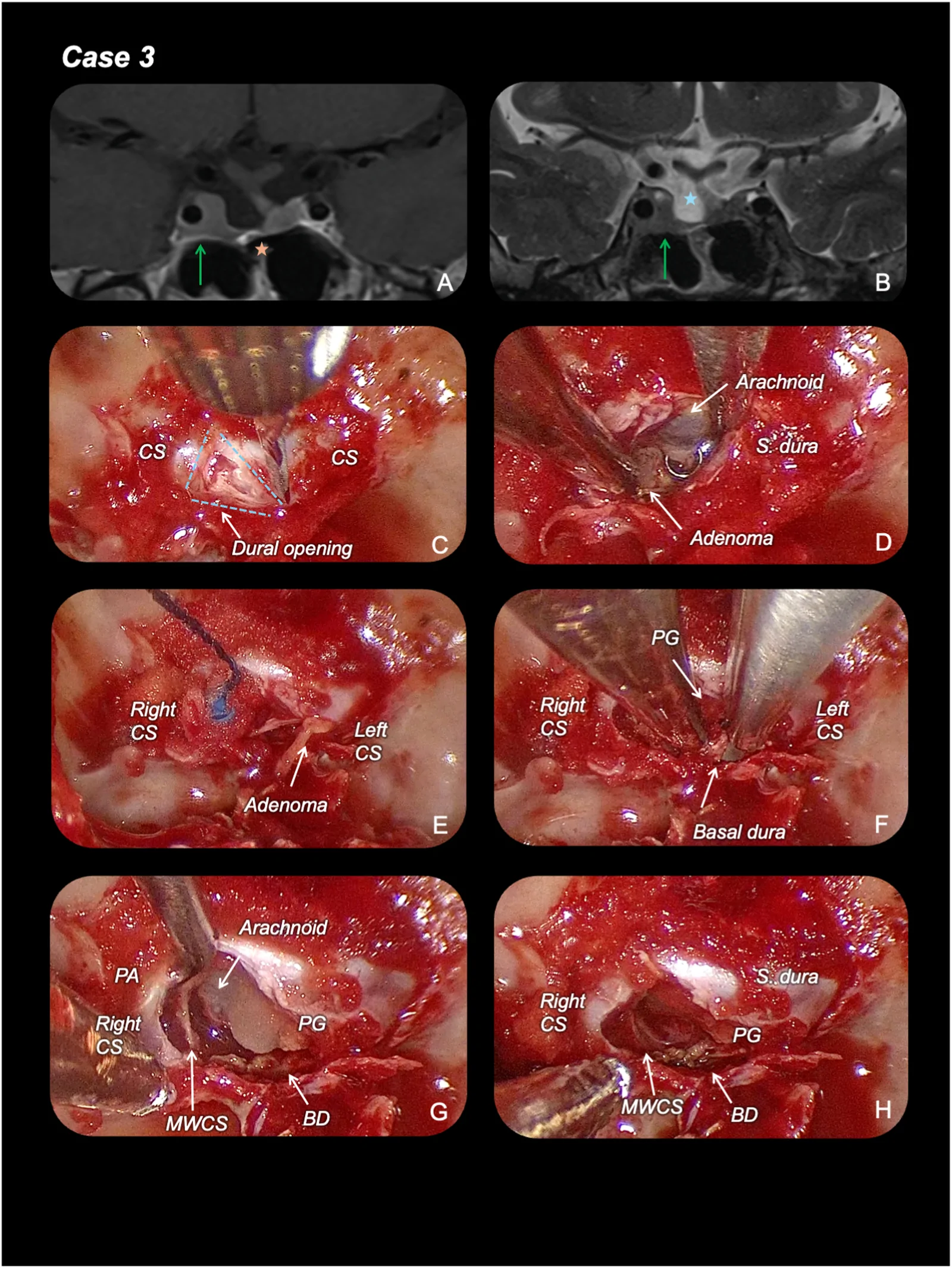
MRI-positive with a macroadenoma, basal dura and cavernous sinus invasion (Case 3) (A–B) Preoperative coronal T1-and T2-weighted MRI showing an 11-mm right-sided adenoma (green arrows), Knosp grade 3B. The orange star indicates basal dura (BD) invasion, while the light-blue star indicates prolapse of the sellar diaphragm. (C–D) Triangular dural opening performed to avoid subarachnoid space opening. Initial partial tumor removal was achieved on the right side. (E–F) During right cavernous sinus (CS) hemostasis, resection of the adenoma and involved basal dura on the left paramedian side. (G–H) Interdural dissection with opening of the medial wall of the cavernous sinus (MWCS), followed by piecemeal intracavernous tumor resection using a curette. Subtotal resection was achieved due to adherence to the right internal carotid artery (ICA). *Abbreviations*. PA: pituitary adenoma; PG: pituitary gland; S.dura: sellar dura.

Intraoperatively, the dura mater was incised in a triangular fashion (Fig. 4, Video 3) to avoid opening of the arachnoid recess. The adenoma was identified on the right side and resected. The medial wall of the cavernous sinus was opened, allowing piecemeal tumor removal. Tumor encasement of the internal carotid artery was confirmed, limiting complete resection due to vascular adherence. In continuity with the invaded basal dura, additional resection was performed on the contralateral side.

No postoperative complications occurred. Despite the absence of postoperative corticotroph insufficiency, the patient achieved eucortisolism and required no adjuvant medical therapy over the 6-month follow-up period. Postoperative MRI demonstrated subtotal resection.

### 3.4 Case 4: MRI-positive with a microadenoma and cavernous sinus invasion

A 41-year-old female presented with an 18-month history of CD. MRI demonstrated a small left-sided posterior and basal microadenoma, with suspected left cavernous sinus involvement. Given the small lesion size, ^18^F-FET PET was performed, confirming left-sided hypermetabolism consistent with the suspected location. Preoperative treatment with osilodrostat was initiated for cortisol control.

**Fig. 5:**
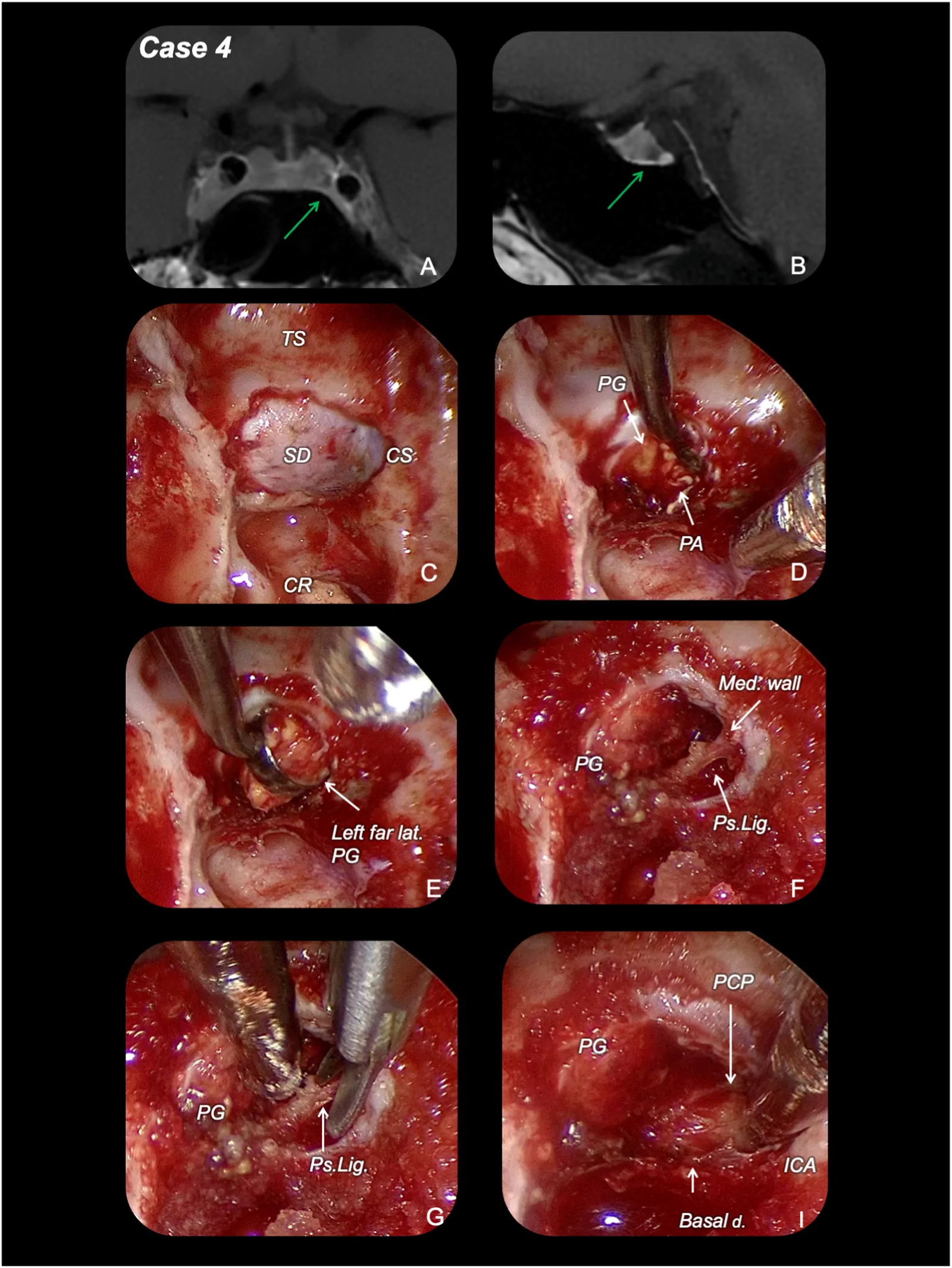
MRI-positive with a microadenoma and cavernous sinus invasion (Case 4) (A–B) Preoperative coronal (A) and sagittal (B) T1-weighted MRI showing a 4-mm left-sided pituitary adenoma (green arrows) with suspected involvement of the cavernous sinus. (C) Endoscopic view after sellar opening showing the sellar dura (SD), tuberculum sellae (TS), cavernous sinus (CS), and clival recess (CR). (D-E) Identification of the pituitary gland (PG) and pathological whitish adenomatous tissue (PA) at the interface between anterior and posterior gland; resection of the far lateral part of the left PG. (F-G) Intraoperative identification of medial wall of the cavernous sinus (Med. wall) invasion and exposure of the parasellar ligament (Ps. Lig.), followed by progressive resection. (G–I) Final aspect of the interdural space between anterior and medial wall of the CS after resection of the PA, showing the surgical cavity, preserved pituitary gland (PG), and exposure of posterior clinoid process (PCP), the internal carotid artery (ICA) and basal dura (Basal d.), with no macroscopic residual tumor.

The adenoma was identified at the interface between the anterior and posterior left pituitary gland and resected (Fig. 5, Video 4). The medial wall of the cavernous sinus was opened, and the invaded portion was removed, given favorable intraoperative conditions. The far lateral left portion of the gland appeared infiltrated and was removed.

No postoperative complications occurred. Postoperatively, early remission was obtained and persisted 8 months after surgery. Postoperative MRI confirmed complete resection.

### 3.5 Case 5: MRI-equivocal with a unifocal heterogeneous signal

**Fig. 6:**
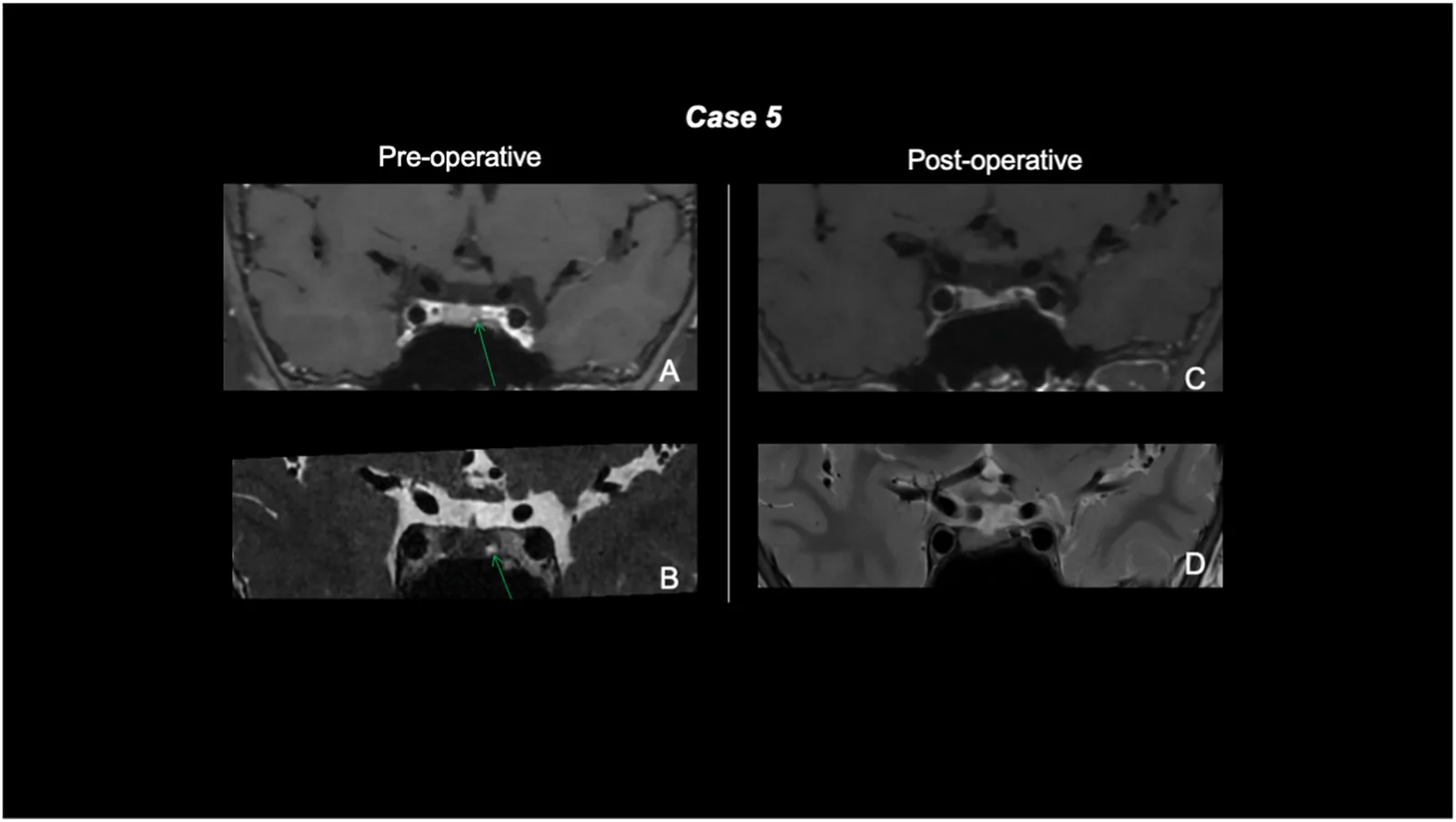
MRI-equivocal with a unifocal heterogeneous signal (Case 5) (A–B) Preoperative MRI (left) showing a 2-mm focal lesion in the left pituitary gland, characterized by spontaneous T1 and T2 hyperintensity (green arrows). Targeted surgical exploration of this heterogeneous signal was decided after multidisciplinary discussion. A tumor was identified and an enlarged adenomectomy was performed, resulting in endocrinological remission. (C–D) Postoperative MRI at 12 months showing no residual tumor.

A 13-year-old male presented with a 4-month history of CD. BMI and growth were controlled without requiring medical therapy. Serial MRI studies performed over a three-year period demonstrated a persistent unifocal heterogeneous T1/T2 signal abnormality within the left pituitary gland. (Fig. 6).

Intraoperatively, a cystic lesion associated with pathological tissue was identified. An enlarged adenomectomy, including a rim of surrounding pituitary tissue was performed.

No residual tumor was observed on postoperative MRI. Histopathological analysis was negative, despite intraoperative identification of abnormal tissue, supporting the possibility of sampling limitations. Endocrinological remission remained stable at 13 months, with clinical improvement and no MRI recurrence.

### 3.6 Case 6: MRI-equivocal with a bifocal heterogeneous signal

**Fig. 7:**
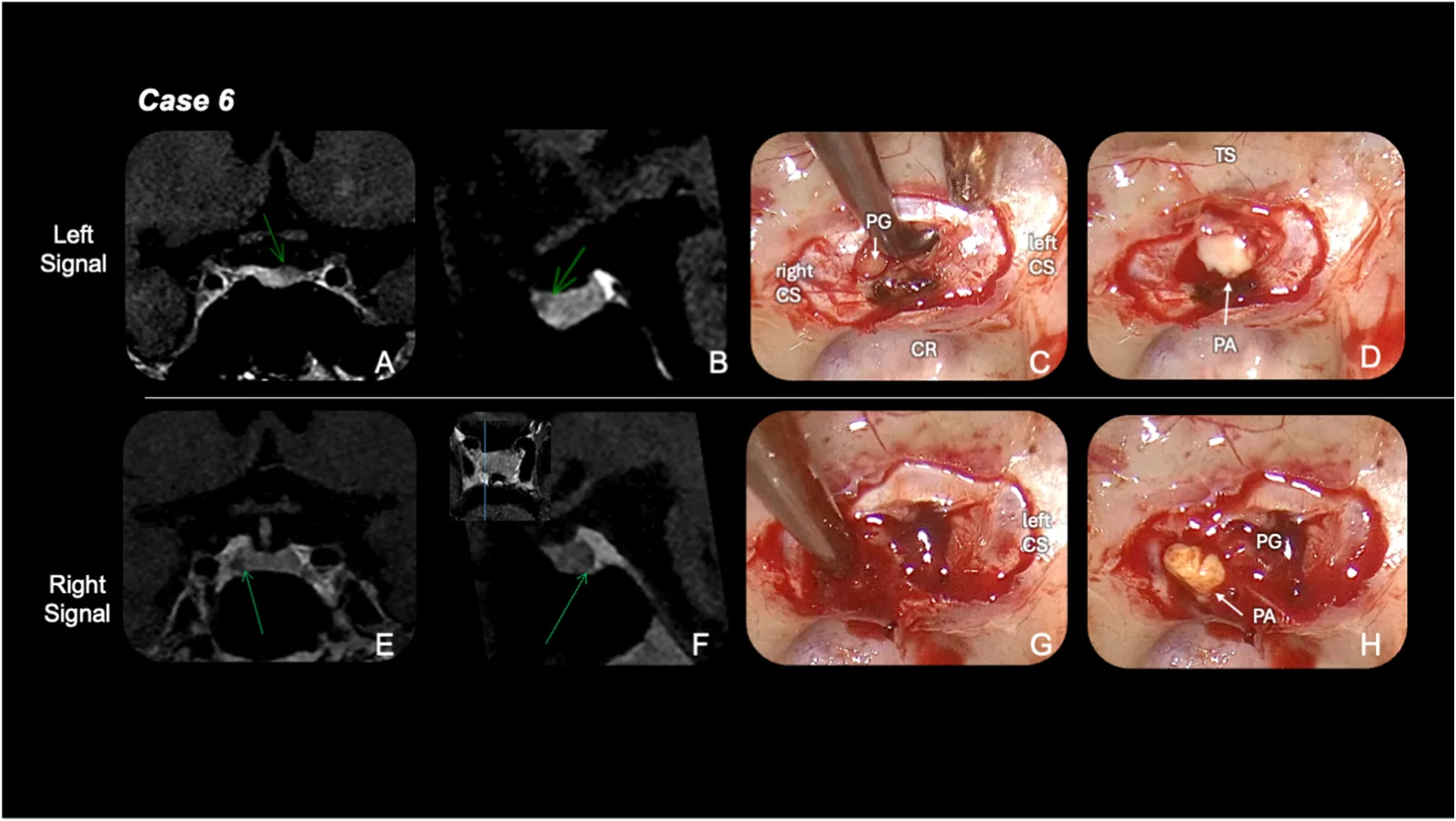
MRI-equivocal with a bifocal heterogeneous signal (Case 6) (A–B) Preoperative MRI showing a 3-mm left-sided focal signal change below the diaphragm, identified on repeat imaging (green arrows). (C–D) Intraoperative targeted left-sided exploration with resection of abnormal whitish tumor tissue, corresponding to the pituitary adenoma. (E–F) Preoperative MRI showing a 3-mm right-sided basal focal signal change, identified on repeat imaging. (G-H) Intraoperative targeted exploration right-sided with identification and resection of abnormal yellowish tissue, corresponding to an agglomerate of Crooke cells on histological analysis.*Abbreviations*: CS, cavernous sinus; PA, pituitary adenoma; PG, pituitary gland.

A 51-year-old male presented with an 11-month history of CD. Serial MRI studies concluded for two equivocal signal abnormalities within the pituitary gland: a left-sided anterolateral hypointense signal and a right-sided posterior paracavernous signal, supporting two potential locations for the corticotroph tumor (Fig. 7, Video 5). IPSS confirmed central origin without clear lateralization.

First, the left anterior lesion located beneath the diaphragmatic recess was explored and resected, appearing as a whitish tissue, distinct from normal pituitary tissue. Second, exploration was extended to the posterior right side, where atypical yellowish pituitary tissue was resected. Histopathological analysis confirmed a corticotroph adenoma in the left-sided specimen, while the right-sided specimen demonstrated aggregates of Crooke cells. No complications occurred. Remission was still achieved 6 months after surgery and no MRI residual tissue was observed.

### 3.7 Case 7: MRI-negative with intraoperative adenoma identification

A 37-year-old female presented with a 30-month history of CD. Repeat MRI did not identify a clear surgical target. IPSS confirmed a central origin but showed no clear lateralization. Medical therapy was initiated (metyrapone followed by osilodrostat), but adequate biochemical control was not achieved.

**Fig. 8:**
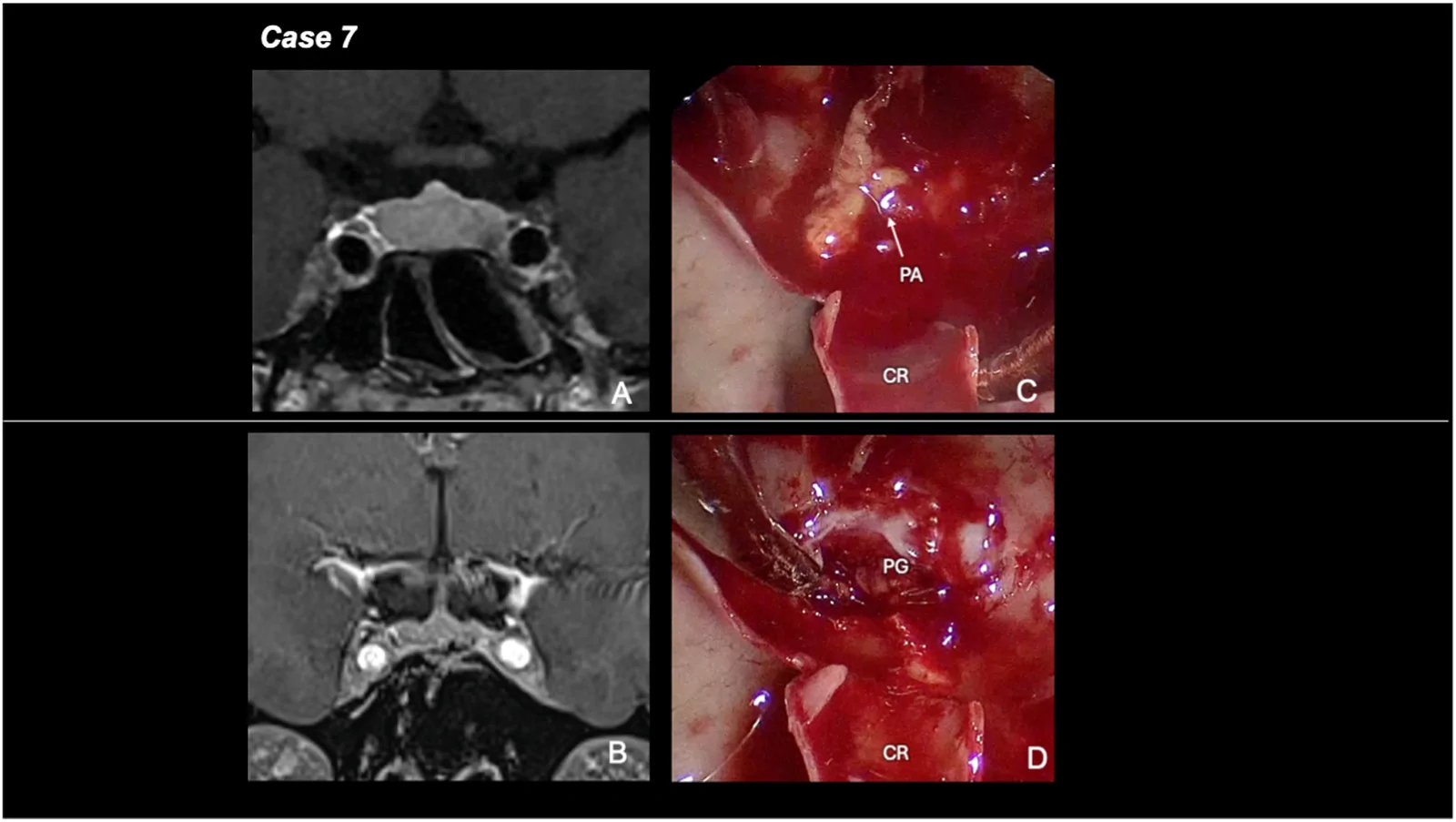
MRI-negative with intraoperative adenoma identification (Case 7) (A) Preoperative coronal T1-weighted MRI showing no identifiable pituitary lesion. (B) Postoperative coronal MRI at 4 months showing no residual. (C) Intraoperative image demonstrating identification of a small right-sided adenoma during pituitary exploration. (D) Intraoperative view of the surgical cavity following enlarged adenomectomy. *Abbreviations*: CR, clival recess; PA, pituitary adenoma; PG, pituitary gland.

Intraoperatively, right-sided exploration identified whitish pathological tissue in the paracavernous region. Enlarged adenomectomy with a margin of adjacent pituitary tissue was performed (Fig. 8, Video 6).

No postoperative complications occurred. Endocrine remission persisted 12 months after surgery. Postoperative MRI showed no residual tumor.

## 4 Discussion

This study illustrates a case-based surgical strategy exploring the spectrum of Cushing's disease, moving beyond the traditional binary classification of MRI-positive versus MRI-negative cases (Yang et al., 2022).

The absence of clear imaging target, as well as lesions with lateral or basal extension, may complicate surgical management and result in lower remission rates (Shapey et al., 2026; Dai et al., 2020). In this context, a clear preoperative strategy is essential to guide intraoperative decision-making. Patients with CD should be managed in high-volume centers by experienced multidisciplinary teams (Fleseriu et al., 2021).

Within this framework, we propose that CD should be considered as a clinical and radiological spectrum rather than a binary entity. Our study aims to illustrate this concept and to present our experience, across different clinical and surgical scenarios.

### 4.1 Preoperative strategy

Cristante et al. (2019) have shown that more than 70% of patients with an initially negative MRI eventually demonstrate a definite or probable abnormality on repeat imaging. As illustrated in Cases 2, 5 and 6, initially negative or equivocal MRI findings do not preclude the presence of a detectable radiological lesion in the future. Importantly, heterogeneous signals abnormalities may represent relevant surgical targets and may guide intraoperative exploration, provided that these signal abnormalities are consistent on multiple MRIs. Advances in imaging techniques, particularly repeated high-resolution MRI, combined with the availability of new steroidogenesis inhibitors (Gadelha et al., 2022), allow a more refined preoperative workup, as illustrated in this series. Effective control of hypercortisolism may improve the patient's clinical condition and, in selected patients, provide additional time to complete the diagnostic workup and obtain repeat pituitary imaging, allowing better characterization of a potential lesion over time. However, this does not establish that medical control improved lesion visibility. Notably, MRI interpretation by expert neuroradiologists was a central component of our algorithm. However, surgery should not be delayed when adequate biochemical control cannot be achieved or when the clinical severity of CD requires prompt treatment.

While IPSS remains the gold standard for confirming a pituitary source of ACTH hypersecretion, its value for tumor lateralization is limited, with mislateralization rates of up to 40% reported in some series (Wind et al., 2013; Lefournier et al., 2003). Current guidelines do not consider IPSS sufficiently reliable for side localization (Reincke and Fleseriu, 2023; Fleseriu et al., 2021; Gadelha et al., 2023; Wind et al., 2013; Estephan et al., 2025). Moreover, correct lateralization does not appear to significantly impact postoperative remission (Wind et al., 2013), and multifocal adenomas may further limit its accuracy (Estephan et al., 2025). Emerging molecular imaging techniques may help overcome these limitations. As illustrated in Case 4, ^18^F-FET PET can improve lesion detection and lateralization in MRI-negative or equivocal cases, with recent studies reporting high sensitivity and potential superiority over IPSS in selected patients (Pruis et al., 2024; Berkmann et al., 2021). Although not yet widely available (Bauman et al., 2023), this technique may represent a promising adjunct in selected cases with small or uncertain MRI targets, although its role in CD remains to be further established.

### 4.2 Surgical aspects and remission rates

The cornerstone of management remains surgical technique and intraoperative identification of the adenoma. In the endoscopic era, improved visualization and magnification are critical advantages (Cappabianca et al., 2004; Caulley et al.). In experienced centers, failure to identify the adenoma intraoperatively becomes increasingly rare (Cristante et al., 2019; Gadelha et al., 2022). When a well-circumscribed lesion is identified, selective resection limited to the adenoma is performed (Cases 1, 2, 3). In contrast, poorly defined lesions require an enlarged adenomectomy including a margin of adjacent normal pituitary tissue (Cases 4, 5, 6 and 7). In MRI-negative cases, partial hypophysectomy may be considered. In our previously published series (Cebula et al., 2017) of 70 MRI-negative patients, partial hypophysectomy was performed in 18.6% of cases, while partial anterior pituitary insufficiency occurred in 4.3%, with no cases of complete anterior pituitary insufficiency. Nevertheless, the risk of pituitary dysfunction should be balanced against the morbidity of persistent uncontrolled hypercortisolism. Of note, histology was negative in case 5, while remission was achieved. In clinical practice, some corticotroph adenomas may be extremely small, sometimes termed picoadenomas, with a fluid consistency, and may be partially or completely aspirated during surgical exploration, resulting in negative histopathological findings.

In cases of dural invasion, particularly involving the cavernous sinus, the bimanual technique through a mononostril endoscopic endonasal approach allows safe opening and resection of the so-called medial wall and increases the likelihood of complete resection. Recent evidence supports this selective approach to obtain better remission rates (Ishida et al., 2022; Cohen-Cohen et al., 2019; Truong et al., 2019; Serioli et al., 2023). Ishida et al. (2022) demonstrated microscopic dural invasion in 57% of cases where it was not apparent intraoperatively, improving the overall RR. However, this step should only be performed under favorable intraoperative conditions and requires substantial expertise. It should therefore be reserved for selected patients and performed by experienced endoscopic skull base teams, given the risk of neurovascular injuries (Cohen-Cohen et al., 2019). Moreover, when basal dura invasion is identified, resection can be safely performed and may contribute to improved remission rates. In a previous study (Baussart et al., 2025a), we reported that an adenomectomy extended to the invaded basal dura achieved short-term remission in 94% of patients, with a significantly higher overall remission rate compared to a control series without systematic dural resection.

This new concept of a CD spectrum may better reflect clinical reality, encompassing clearly visible adenomas, heterogeneous signals, and MRI-negative cases requiring exploration. It also accounts for the dynamic nature of imaging findings and their potential discordance with biochemical data, allowing a more flexible and individualized surgical approach. Our outcomes are consistent with previously reported low complication and remission rates. With the current evolution of CD surgery, patient outcomes have improved, as mainly reported (Fleseriu et al., 2021; Gadelha et al., 2023; Karsy et al., 2025). Consequently, remission in grade 1 to 3A tumors according to the revised Knosp classification (Micko et al., 2015) can be achieved in selected patients. However, the management of grade 3B and 4 adenomas (Case 3) remains a major challenge (Estephan et al., 2025; Micko et al., 2020). In such cases complete resection is often precluded by vascular adhesions, resulting in lower remission rates and frequent need for adjuvant therapy (Dai et al., 2020; Stroud et al., 2020).

### 4.3 Limitations

The main limitations of this study include its illustrative design, small sample size, relatively short follow-up, and absence of a comparator group. The retrospective, non-consecutive selection of cases after outcomes were known introduces an inherent risk of selection bias, while the evolution of surgical practice over time and the partly subjective nature of MRI classification may further limit interpretation. Therefore, the reported outcomes should not be considered representative of the entire institutional cohort, and the proposed framework may have limited generalizability outside high-volume expert centers. Further evaluation in a consecutive prospective cohort is required to assess its impact on surgical and endocrinological outcomes.

## 5 Conclusions

Cushing's disease should be approached as a spectrum of anatomical, radiological, and surgical situations rather than a binary condition defined by MRI status. Our case-based algorithm provides a structured, flexible, and individualized framework integrating imaging, medical therapy, and intraoperative findings to guide surgical decision-making across this spectrum. Its impact on surgical outcomes requires further evaluation.

## Ethics declaration

Written informed consent to take part in the study and to publish the article has been obtained from all participants or their legal representatives. The privacy rights of participants have been observed.

This study was performed in compliance with relevant laws, regulatory frameworks and guidelines where the research took place. Ethics committee approval was not required under relevant laws and institutional guidelines. According to French regulations (Loi Jardé), this retrospective study did not require approval by a Comité de Protection des Personnes (CPP)

## Informed consent statement

Informed consent was obtained from all subjects involved in the study.

## Data availability statement

Data supporting the findings of this study are available from the corresponding author upon reasonable request.

## Funding

This research received no external funding.

## Declaration of competing interests

The authors declare that they have no known competing financial interests or personal relationships that could have appeared to influence the work reported in this paper.
